# DNA-Mediated Carbon Nanotubes Heterojunction Assembly

**DOI:** 10.1021/acsnanoscienceau.4c00025

**Published:** 2024-09-06

**Authors:** Zechariah Mengrani, Weiying Hong, Matteo Palma

**Affiliations:** Department of Chemistry, Queen Mary University of London, London E1 4NS, U.K.

**Keywords:** single-walled carbon nanotubes (SWCNTs), DNA, chirality, heterostructures, molecular junctions

## Abstract

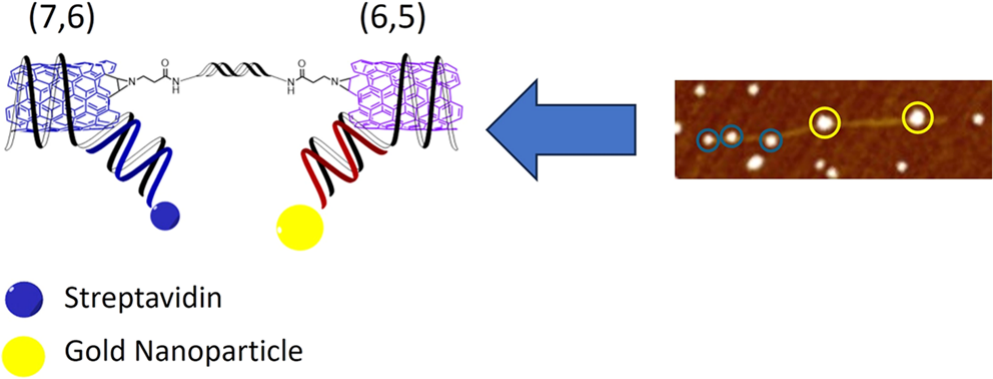

Herein, we present a strategy for the controlled assembly of single-walled carbon nanotube (SWCNT) linear junctions mediated by DNA as a functional linker. We demonstrate this by employing SWCNTs of two different chiralities via the specific design of DNA sequences and chiral selection. Streptavidin and AuNP labeling of the SWCNT sidewalls demonstrate the presence of two different chirality within each individual CNT–DNA–CNT junction. These one-dimensional nanohybrids were further organized from solution to devices. The approach we developed is of general applicability for the assembly of functional nanohybrids based on carbon nanotubes toward functional applications.

Single-walled carbon nanotubes (SWCNTs), thanks to their nanoscale nature and electrical, mechanical, and thermal properties, hold promise as candidate materials for electronic systems and devices beyond the limits of the silicon-based technology.^1−4^ In this context, junctions among SWCNTs are of interest for the development of (molecular) electronic devices, biosensors, and nanomechanical systems.^5−15^ In particular, there is an interest in using SWCNTs as nanoelectrodes due to their natural compatibility with different moieties, easy processability, and nanoscale size.^16−21^

In this context, SWCNTs have been interconnected via chemical functionalization to form junctions employing a variety of strategies and molecular linkers.^22−27^ In order to form end-to-end junctions in solution and ensure reactions are targeted toward the ends of SWCNTs, Swager et al. employed a side-wall protection step before regio-specific covalent functionalization between two nanotubes using an oligonucleotide.^28^ Furthering upon this, we demonstrated the formation of CNT junctions in aqueous solution employing a DNA wrapping strategy to protect the sidewalls.^24−26^ The interaction between the DNA and the sidewalls of the nanotubes further enables dispersion in solution, alongside enabling the separation of nanotubes of different chirality.^29^

The controlled assembly of linear end-to-end junctions through in-solution processes is very desirable for the low-cost fabrication of molecular electronic systems and devices^25,27,30,31^ and improved charge injection from tube to tube in networks with enhanced electric conductivity. Notably, the ability to control in-solution junction formation among SWCNTs of different chirality can enable the facile assembly of intrinsic nanoscale diodes.^32,33^

Among the various functional moieties that have been used to link SWCNTs,^24−27^ DNA has proven to be a versatile linker moiety. DNA can indeed be incorporated into various nanostructures as a building material.^34,35^ In this context, we have employed DNA as a stimuli-responsive DNA–CNT and DNA–MoS_2_ linker, taking advantage of the control that can be achieved with DNA depending on the environment it is subject to.^26,36^ Moreover, we employed DNA to tune the assembly of single-molecule heterostructures linking quantum dots and proteins to SWCNTs.^37,38^

Herein, we present a strategy (Scheme 1) employing DNA for the controlled assembly of linear end-to-end SWCNT junctions from two different chirality nanotubes. To demonstrate the formation of junctions made of different chirality SWCNTs, a labeling strategy was employed. Streptavidin was used to tag the sidewall of (7,6) SWCNTs while gold nanoparticles (AuNPs) were tethered to (6,5) SWCNTs. The ability to link SWCNTs of different chirality, and hence electronic character, can be advantageous in the ad hoc design of SWCNT junctions as nanoscale diodes for future implementation in nano- and molecular electronic systems that could show potential quantum size effects.^32^ To the best of our knowledge, this is the first demonstration of solution-processable carbon nanotube junctions of distinct chirality, demonstrated with single nanostructure control and employing DNA as a linker.

**Scheme 1:**
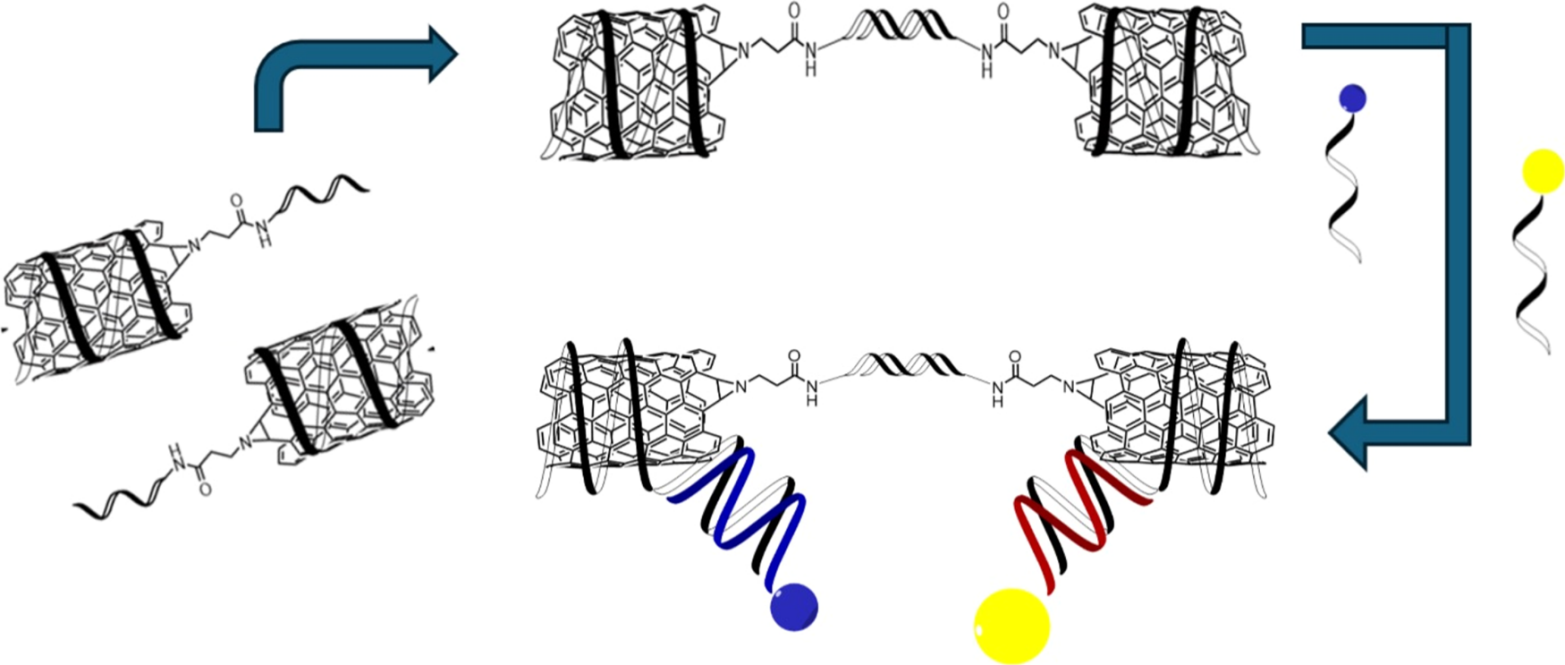
Formation of Particle-Labeled SWCNT Junction Blue circles represent streptavidin particles, yellow circles represent AuNPs.

## Results and Discussion

(6,5)- and (7,6)-enriched carbon nanotubes were dispersed in aqueous solution via DNA wrapping (see the Materials and Methods section and the Supporting Information (SI)), which protected the sidewalls of the SWCNTs, leaving mostly the nanotube termini available for further functionalization.^29^ The ultraviolet (UV) spectra demonstrating the chirality of the nanotubes used can be seen in Figures S1 and S2. (6,5) SWCNTs were separated via an aqueous two-phase extraction method (Figure S2 demonstrates the red shift of the DNA exchange procedure);^39,40^Figure S3 shows a comparison of the two chirality used, while Figure S4 displays the presence of both chirality CNTs within the junctions solution.

**Figure 1:**
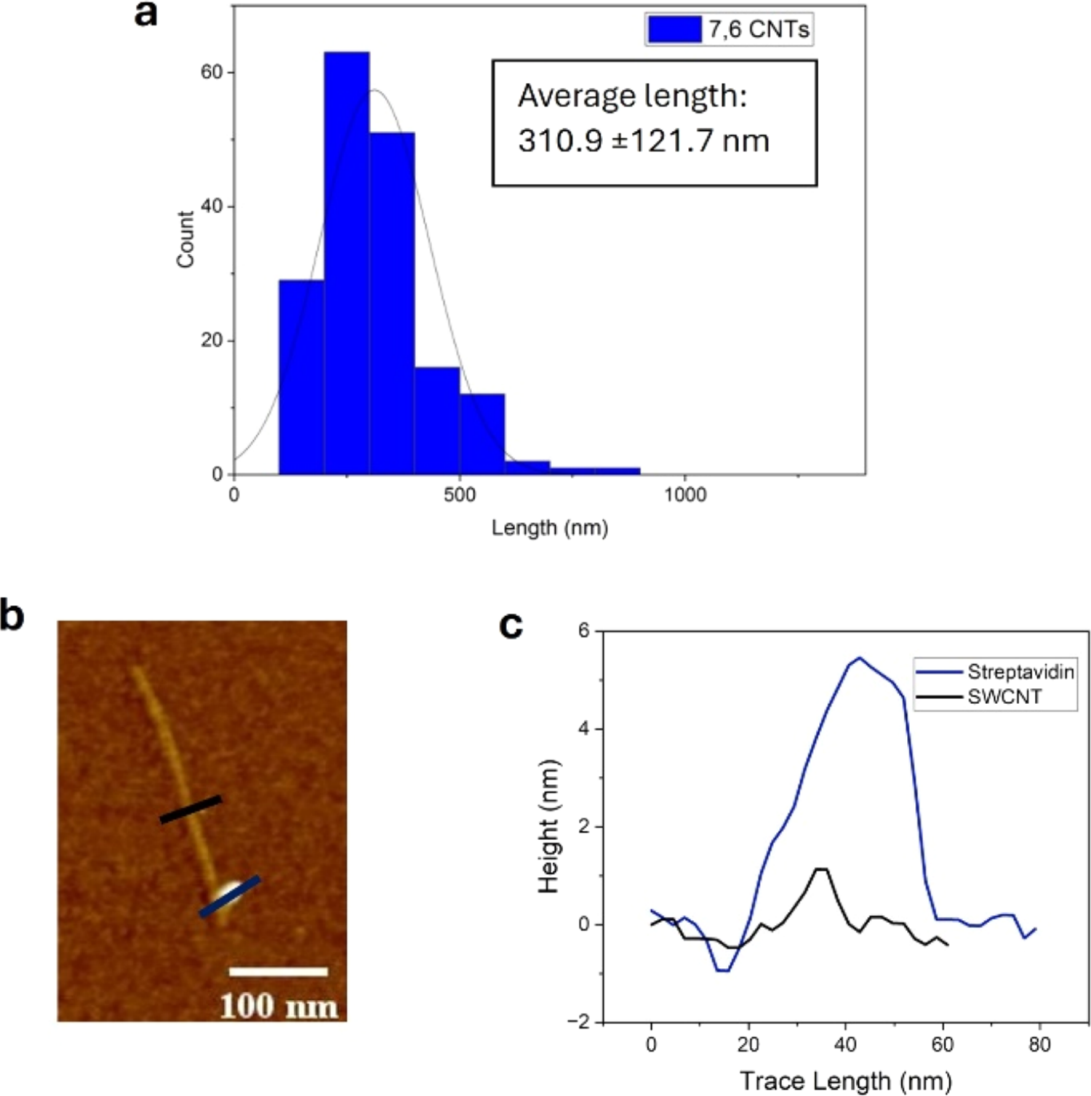
(a) Histogram displaying the length distribution with a fitted curve of DNA-wrapped^6,7^ SWCNTs; (b) AFM image of streptavidin-labeled (7,6) SWCNT; (c) height profile comparing the streptavidin ca. 5 nm (blue) and the (7,6) SWCNT ca. 1 nm (black).

Characteristic atomic force microscopy (AFM) images of DNA-wrapped SWCNTs drop cast on mica can be seen in Figure S5a,b. Pristine (7,6) DNA-wrapped SWCNTs were statistically analyzed via AFM, with an average length of 310.9 ± 121.7 nm. The length distribution of these SWCNTs can be seen in the histogram in Figure 1a (corresponding AFM images are seen in Figure S5a).

To label (7,6) chirality-enriched SWCNTs, we utilized the strong interaction of complementary DNA. The attachment of the particle occurred through the hybridization between streptavidin-conjugated biotinylated DNA and DNA-wrapped (7,6) SWCNTs.^41^ The procedure for this hybridization can be seen in the SI (Scheme S1). This double-stranded DNA (dsDNA) formation leads to the particle attachment on the SWCNT. To form this hybrid structure of streptavidin-labeled (7,6) SWCNTs, we mixed the streptavidin-conjugated biotinylated DNA with the DNA-wrapped SWCNTs. Figure 1b shows a representative AFM image of this hybrid alongside the respective height profiles corresponding to streptavidin (ca. 5 nm) and SWCNT (ca. 1 nm). The difference between the height of the SWCNT (black) and the streptavidin attached (blue) can be seen in Figure 1c.

**Figure 2:**
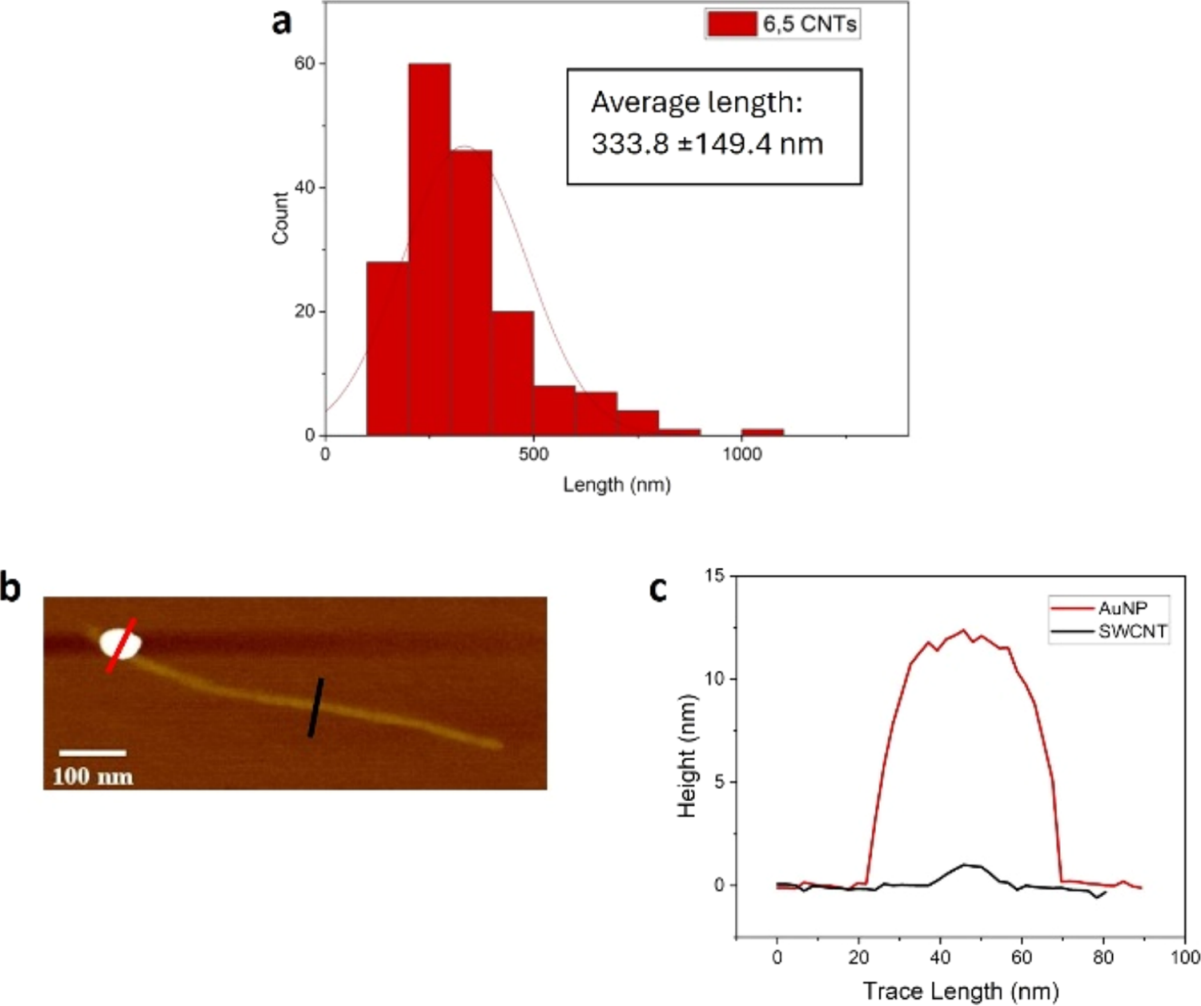
(a) Histogram displaying the length analysis done for pristine DNA-wrapped (6,5) SWCNTs; (b) AFM image of AuNP-labeled (6,5) SWCNT; (c) height profile comparing the AuNP ca. 10 nm (dark red) and the (6,5) SWCNT ca. 1 nm (black).

Pristine (6,5) DNA-wrapped SWCNTs were statistically analyzed via AFM and were shown to exhibit an average length of 333.8 ± 149.4 nm. The length distribution of these SWCNTs can be seen in the histogram in Figure 2a (with the corresponding AFM images seen in Figure S5b).

A similar DNA hybridization strategy was used for (6,5) SWCNTs. Attaching the AuNP occurred through the hybridization between AuNP-conjugated thiolated DNA and DNA-wrapped (6,5) SWCNTs.^42^ The procedure for this hybridization alongside the DNA strands used can be seen in the SI (Scheme S2). Similarly, with the streptavidin labeling, the wrapping strands on the SWCNT partially complement the ssDNA attached to the AuNP. To form the hybrid structure of AuNP-labeled (6,5) SWCNTs, the DNA-functionalized AuNPs were mixed in solution with the DNA-wrapped SWCNTs. The nanohybrid structure of AuNP-labeled (6,5) SWCNTs was shown in AFM images (Figure 2b and more representative AFM image in Figure S6) with the respective height profiles (Figure 2c) corresponding to the AuNP (ca. 10 nm) and the SWCNT (ca. 1 nm).

**Figure 3:**
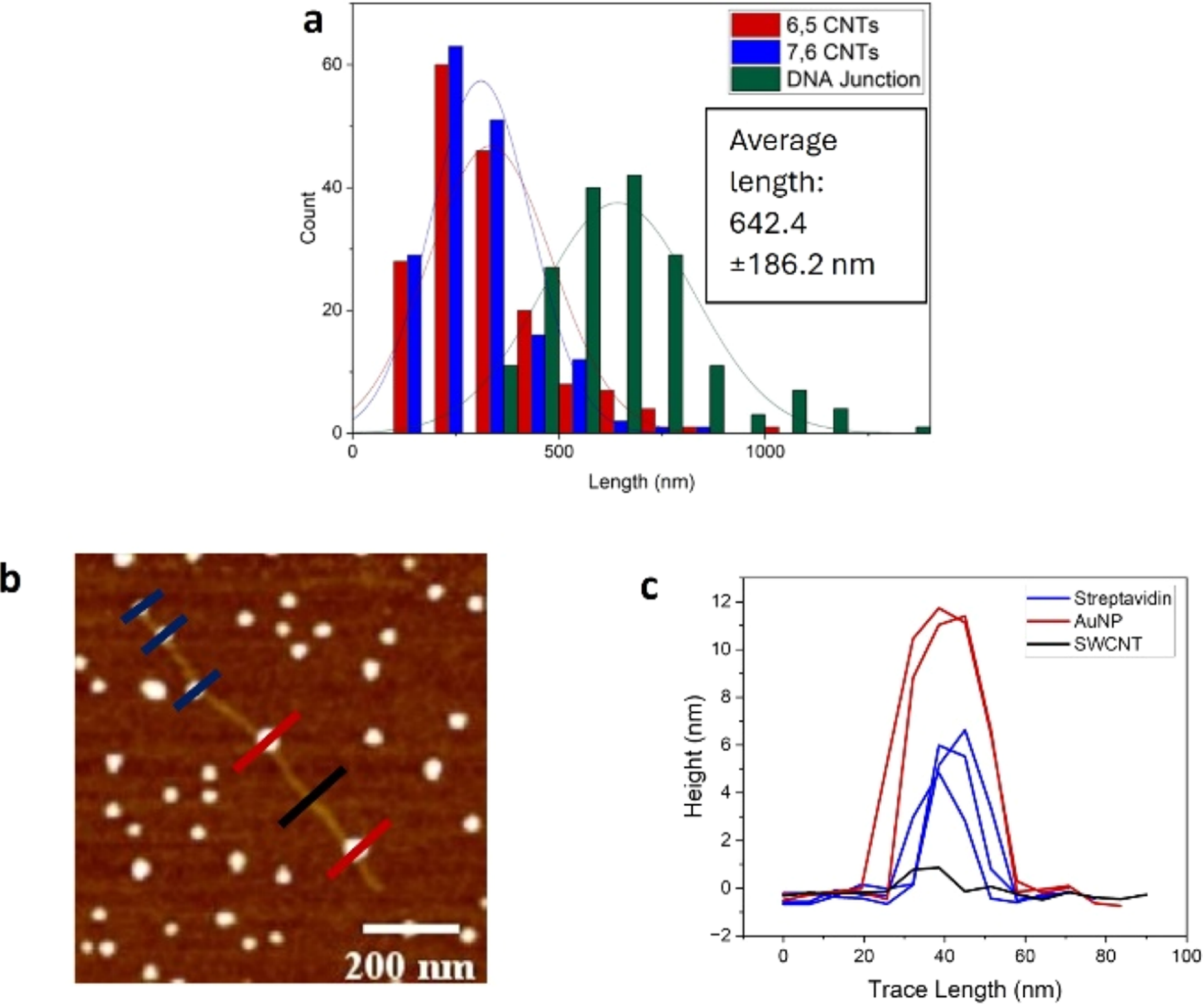
(a) Histogram displaying the length analysis done for the mixed-chirality junctions formed; (b) nanoparticle labeling of mixed-chirality junction; (c) height profile analysis comparing the various nanoparticles streptavidin ca. 5 nm (blue), gold nanoparticle ca. 11 nm (dark red), with the CNTs of ca. 1 nm (black).

For the formation of junctions, we adapted a previously utilized methodology.^26,38^ This involved using azide-functionalized ssDNA to react with the ends of the SWCNTs: the solutions were UV-irradiated with a medium pressure immersion mercury lamp (Photochemical Reactors Ltd.) to promote the formation of reactive nitrene groups, which in proximity of the free-SWCNT-tips form aziridine photoadducts by a cycloaddition reaction (see the Materials and Methods section alongside the SI, Scheme S3). Each enriched chirality SWCNT was conjugated to the corresponding DNA strand to form a complementary dsDNA junction for hybridization. An increase of the D/G ratio in the Raman spectra of the CNTs after functionalization, for each chirality CNT and the final mixed-chirality junction, suggests successful functionalization of the CNTs by DNA (Figure S7). Diluted solutions of assembled SWCNT junctions were cast on mica and characterized via AFM. A statistical comparison between pristine nanotubes and formed junctions was conducted, and Figure 3a shows the length distribution of the CNT junctions in comparison to the pristine nanotubes, in which the junctions exhibit an average length of 642.4 ± 186.2 nm (see Figure S8 for corresponding AFM images of these junctions). This increase of almost double the length of the pristine starting carbon nanotubes strongly suggests junction formation.

Once junction formation had been confirmed, the labeling of the different chirality within the junction was done by following the procedure: mixing the ssDNA-functionalized particles (streptavidin and AuNPs) with the SWCNT junction solution and incubating overnight. The solution was then cast on mica and characterized via AFM.

The particle attachment can be seen in Figure 3b, with two kinds of particle (streptavidin and AuNP) labeling alongside a single SWCNT junction observed in the AFM image. In Figure 3c, the blue lines indicate streptavidin particles, the dark red lines indicate AuNPs, and the black line represents the SWCNT. Two distinct regions of labeling on a single SWCNTs junction are evident, with streptavidin concentrated at one end and AuNPs at the other, with the streptavidin displaying a height of ca. 5 nm and the AuNP showing a height of ca. 11 nm, as expected. This indicates a clear (7,6)–(6,5) junction orientation from left to right. Larger representative AFM images of this labeling can be seen in Figures S9 and S10, with more examples of these labeled junctions clearly seen in Figures S11 and S12. A 38% yield of labeled CNT junctions was determined from AFM images and height profiles.

**Figure 4:**
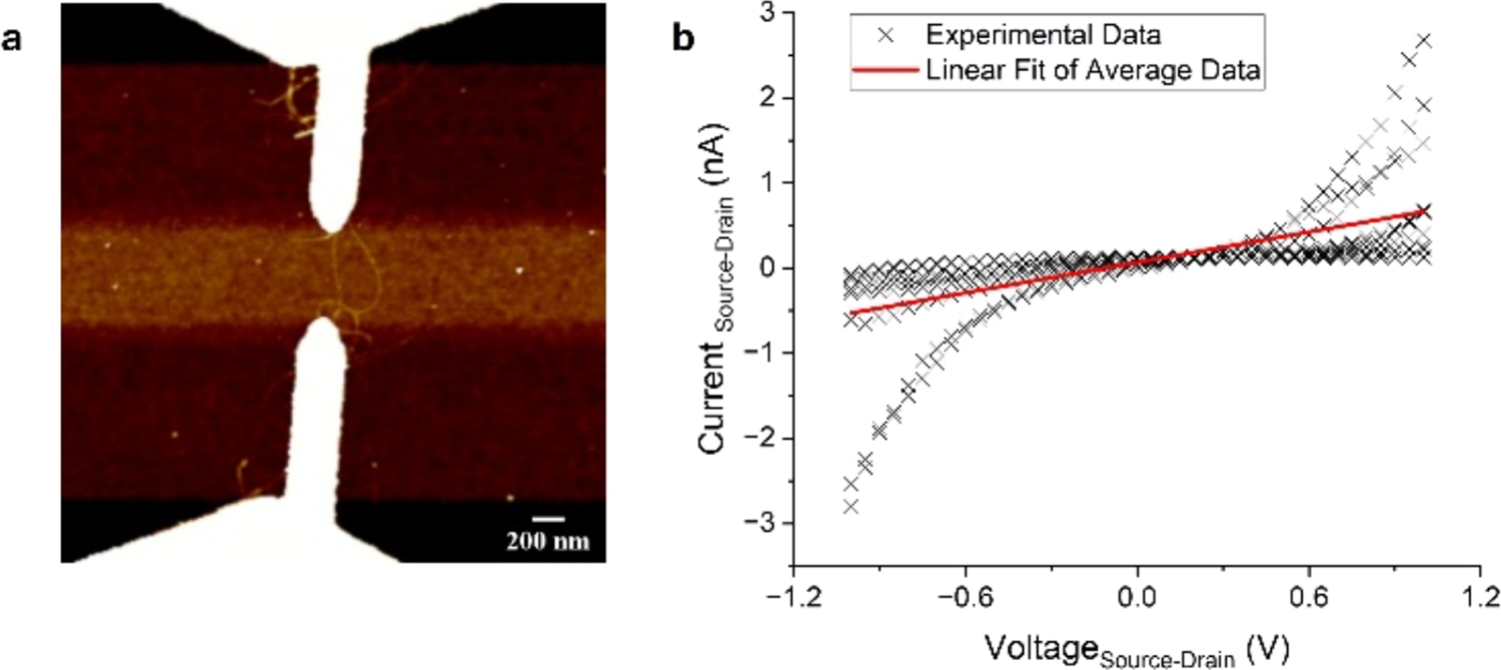
(a) AFM of 300 nm electrode pads bridged by a mixed-chirality junction; (b) *IV* graph of current flow from 9 devices with a linear fitting displaying ohmic behavior of mixed-chirality junction.

To investigate the electrical properties of this hybrid structure, we measured the current–voltage characteristics of DNA-linked mixed-chirality CNTs (Scheme S3) in a device configuration. The immobilization of the junction was achieved by utilizing a surface chemistry modification displayed in Liu et al.,^27^ where gold electrodes on a prepatterned silicon chip were functionalized using cysteamine to form self-assembled monolayers. The CNT junctions were drop cast on these prepatterned and functionalized patterned substrates, forming amide bonds that randomly create bridges between the electrodes. Nine devices were successfully immobilized using this method out of a total of 32, resulting in a 28% yield. Figure 4a displays the successful bridging of two electrodes with a mixed-chirality junction. Once the electrodes had been bridged by the junction, electrical measurements were taken. A simple voltage bias from −1 to +1 V was applied to the immobilized junction. Figure 4b displays the average data collected from 9 electrode pads that were successfully bridged. It can be seen that a linear relationship is observed, demonstrating the ohmic nature of the CNT–DNA–CNT junction. These devices are expected to exhibit a linear ohmic relationship, as seen in Stern et al., where a voltage bias resulted in a linear relationship when measuring current through junctions in a SWCNT network.^43^

From the current–voltage data obtained, the average conductance of these devices was calculated to be 7.69 ± 7.94 × 10^–6^*G*_0_ (where *G*_0_ is the conductance quantum, *G*_0_ = 77.48 μS): see Materials and Methods section. Single-molecule break-junction-based conductance measurements have been done on 12-base pair (bp) DNA, displaying values on the order of magnitude 10^–4^*G*_0_.^44^ However, Liu et al.^27^ electrically characterized CNT–DNA–CNT junctions using DNA of lengths ranging from 9 to 18 bp and obtained conductance values in line with our measurements for their longer DNA linkers.^27^ The resistance of our system was calculated to be 1.68 ± 5.32 GΩ. This value is higher than previously reported for nanopatterned DNA–CNT junctions, where the DNA was linked via the formation of an amide bond on cut CNT termini.^20^ It should be noted that the lengths of the DNA used, and the linker chemistry differed; hence, the variation in the conductance values are unsurprising.^45^ By and large, we can consider our conductance values to be reasonable for our type of single-molecule CNT–DNA junction.

## Conclusions

This work focused on creating solution-processable linear end-to-end molecular transport junctions using different chirality SWCNTs and DNA in an aqueous environment. DNA was employed to protect the sidewalls of the nanotubes, promote their end functionalization, and act as a molecular linker. By using complementary ssDNA, two different chirality SWCNTs were functionalized with DNA in solution via a UV-promoted cycloaddition reaction and then successfully joined together via hybridization. In order to verify junction formation, streptavidin (ca. 5 nm) was attached to (7,6) SWCNTs, while AuNPs (ca. 10 nm) were employed to label (6,5) SWCNTs; the subsequent identification of the junction was obtained via AFM imaging with distinct regions in which streptavidin and AuNPs are concentrated at opposite parts of a single SWCNT junction. The junctions were further immobilized onto devices, and current–voltage electrical characterizations were performed. The measured resistance and conductance behavior broadly aligned with the existing literature investigating DNA single-molecule junctions. The method presented here can conceivably allow the fabrication of nanoelectronic systems based on SWCNTs of selected chirality toward the potential formation of tailored bioelectronic devices and nanoscale intrinsic diodes upon tuning the semiconducting nature of the nanotubes and the chemical nature of the linking moiety.

## Materials and Methods

### Materials

(6,5)- and (7,6)-enriched SWCNTs were purchased from Sigma-Aldrich; all DNA oligonucleotides, including sequences with Azide functionalization (6 carbon alkyl chain, molecular weight of 318.3 g/mol) on the 5′ end of the DNA were purchased from Integrated DNA technologies. Streptavidin (ca. 5 nm) and AuNPs (ca. 10 nm) were purchased from Sigma-Aldrich. Substrates used for the electrical characterization were purchased from ConScience with specifications of 300 nm gap electrode spacing, with 5 nm Titanium (Ti)/45 nm Gold (Au) on a 4 in. doped (100)-Si Wafer with 400 nm thermal oxide.

### 7,6 SWCNTs Wrapping

(7,6)-enriched SWCNTs were used as purchased to form a solution of 1 mg/mL DNA-wrapped SWCNTs. One milligram of SWCNTs was mixed with a 1 mL aqueous solution of DNA (2 mg/mL ssDNA, 0.1 M NaCl) via bath sonication in ice water for 90 min. The wrapping DNA strand is composed of two domains from the 5′ to the 3′ end: one domain has affinity for the (7,6) SWCNT, and the other domain is complementary for streptavidin labeling. The solution was centrifuged at 15,060 rpm for 60 min, and the supernatant was carefully pipetted out and stored in a microcentrifuge tube.

### 6,5 SWCNTs Wrapping

6,5 chirality pure SWCNTs were extracted from an aqueous two-phase extraction method used by Li et al.^39^ before a DNA exchange procedure was done following the methodology from Streit et al.^40^ 150 μL portion of purified (6,5) SWCNT in 1% aqueous sodium deoxycholate (DOC), 25 μL of 25% polyacrylamide (PAM), and 30 μL of 10 mg/mL DNA was mixed. 90 μL of methanol was added drop by drop, 3 times to a total of 270 μL. 600 μL of IPA was then added to the mixture and vortexed. The solution was then centrifuged at 15,060 rpm for 2 min with a low deceleration rate. The supernatant was removed, and the pellet was redispersed in 1× tris/borate/EDTA (TBE) buffer for a total solution of 150 μL. To ensure the removal of the DOC, the addition of IPA to the dispersal step was repeated up to 3 times. The solution was then dialyzed against MiliQ H_2_O for 2 h to remove excess DNA.

### Assembly of Mixed-Chirality Junction

The functionalization of SWCNTs using azide-modified DNA and formation of the mixed-chirality junction used a combination of methodologies published by Amoroso et al.^26^ and Freeley et al.^38^ SWCNTs were mixed with azide-modified DNA (250 nM), 2.5 μL of 10× Dulbecco’s phosphate-buffered saline (DPBS), and made up to a final volume of 25 μL with MiliQ H_2_O. This solution was exposed to UV light (400 W) for 20 min and then placed on a shaker for 1 h at room temperature. Dialysis against 1× DPBS with Milipore dialysis membranes (20 kDa MWCO) was done to remove excess DNA before normalizing the solutions to 50 μL with 1× DPBS. To form the mixed-chirality junction, the two chirality of azide-functionalized SWCNTs were mixed and incubated overnight on a shaker at room temperature.

### Nanoparticle Labeling

The functionalization of nanoparticles was performed first before combining them with their respective SWCNTs. The functionalization of the streptavidin nanoparticle occurred through its strong interaction with biotin.^41^ It was done by mixing streptavidin nanoparticles in equimolar amounts (4 nM) with biotin-modified DNA and incubated for 5 min. The solution was then stored in the refrigerator for further use. To label the (7,6) SWCNTs, 1 μL of (7,6) SWCNTs was diluted 5× with 1× TAE buffer (40 mM Tris, 20 mM acetic acid, 1 mM EDTA, and 12.5 mM MgCl_2_) before being incubated with 1.5 μL of functionalized streptavidin for 5 min at room temperature.

The functionalization of the AuNP utilized the interaction between the thiol-modified DNA and the AuNP.^42^ The nanoparticle was functionalized by adapting the methodology presented by Xu et al.^46^ 0.4 μL of 10% Tween 80 was mixed with 100 μL of AuNP and left incubating for 30 min at room temperature. One microliter of thiol-modified DNA (100 μM) and 20 μL of tris(2-carboxyethyl)phosphine (TCEP) (0.5 M, 2.8 mg in 20 μL) were added, and the solution was left to incubate at 50 °C for 2.5 h. To remove the excess DNA, filter centrifugation was done using Millipore centrifuge filters (Amicon ultra 0.5 100 kDa) for 3 min at 8000 rpm washed by DPBS. This was repeated 3 times. To tag the (6,5) SWCNTs with the functionalized AuNPs, 10 μL of (6,5) SWCNTs diluted 100× with 1× Tris buffer (50 mM Tris, 20 mM KCl) was incubated with 1 μL of functionalized AuNPs in the fridge overnight.

Labeling of the junction solution occurred by incubating 5 μL of 5× diluted junction solution with 0.1 μL of functionalized AuNPs and 1.5 μL of functionalized streptavidin and incubating overnight in the fridge.

### Atomic Force Microscopy

Analysis of the SWCNT samples was done through atomic force microscopy (AFM) on a Bruker Dimension Icon in PeakForce Tapping mode with ScanAsyst Air tips from Bruker. Samples were deposited onto freshly cleaved mica disks. Five microliters of the sample was deposited and left incubating on a shaker for 10 min. They were then blow-dried using Ar (Ar) gas before washing with 20 μL of MiliQ H_2_O and dried again.

Nanoscope Analysis software was used to analyze the AFM images. This involved flattening the images alongside normalizing the *Z*-scale. The cut tool was used to analyze structure heights from which height profiles were obtained.

### Electrical Characterization of Mixed-Chirality SWCNT Junction

Prepatterned Silicon chips were used to test the electrical characteristics of the mixed-chirality junction. To immobilize the junctions onto the electrode pads, a surface functionalization was done using a methodology by Liu et al.^27^ The silicon chip was prepared by first bath sonicating the chip in acetone and Isopropanol (IPA) for 15 min each before being subject to plasma treatment for 5 min. After the chip was washed with MiliQ H_2_O, it was immersed in cysteamine (10 mM) solution overnight. The chip was washed and dried before 5 μL of junction solution mixed with hexafluorophosphate azabenzotriazole tetramethyluronium (HATU, coupling agent, 0.13 mM) in DBPS buffer was drop cast onto the silicon chip and incubated at room temperature on a shaker for 2 h. After incubation, the chip was washed with 200 μL of MiliQ H_2_O and dried via Ar.

For the electrical characterization, a probe station (PS-100, Lakeshore) equipped with a semiconducting parameter analyzer (Keithley, 4200SCS) was used, and the measurements were done at room temperature. To measure the current through CNT–DNA-CNT devices as a function of voltage (*I*–*V* curves), a sweeping bias (−1–1 V) was applied between the source and drain electrodes.

### Conductance Calculations

Conductance calculations were done in reference to the equation *G* = *I*/*V* with *G* = Conductance; I = Current; *V* = Voltage. The value *G* was obtained from the current–voltage graph, in which the change of current was divided by the change of voltage before being divided by the conductance quantum of *G*_0_ (77.48 μS).^47^ The resistance of this system can be calculated from the inverse of the conductance calculated. Resistance is calculated by *R* = 1/*G*.

## Supporting Information Available

UV absorption spectra of (7,6), (6,5) SWCNTs and junction solution; AFM images of SWCNT solutions; schemes of particle labeling; AFM of AuNP labeling; Raman spectra of individual chirality SWCNTs and junction solution; AFM of junction solution; large area AFM images of junction labeling, alongside more individually labeled junctions (PDF)

## Abbreviations

SWCNT: single-walled carbon nanotube
DNA: deoxyribonucleic acid
AuNPs: gold nanoparticles
AFM: atomic force microscopy
ssDNA: single-stranded DNA
dsDNA: double-stranded DNA
bp: base pair
