# Supplementary material for: DNA-Mediated Carbon Nanotubes Heterojunction Assembly
Source: ACS Nanosci Au. 2024 Sep 6;4(6):391–8. doi: 10.1021/acsnanoscienceau.4c00025 (PMC11659895; doi:10.1021/acsnanoscienceau.4c00025)
Supplement: Supplementary file 1 — ng4c00025_si_001.pdf [file ng4c00025_si_001.pdf]

Supporting Information

DNA-Mediated Carbon Nanotubes Heterojunction Assembly

Zechariah Mengrani [1+], Weiying Hong[1+], Matteo Palma[1*]

[1] Department of Chemistry, Queen Mary University of London, London E1 4NS, UK

[+]These authors contributed equally to this work.

[*]Corresponding Author: m.palma@qmul.ac.uk

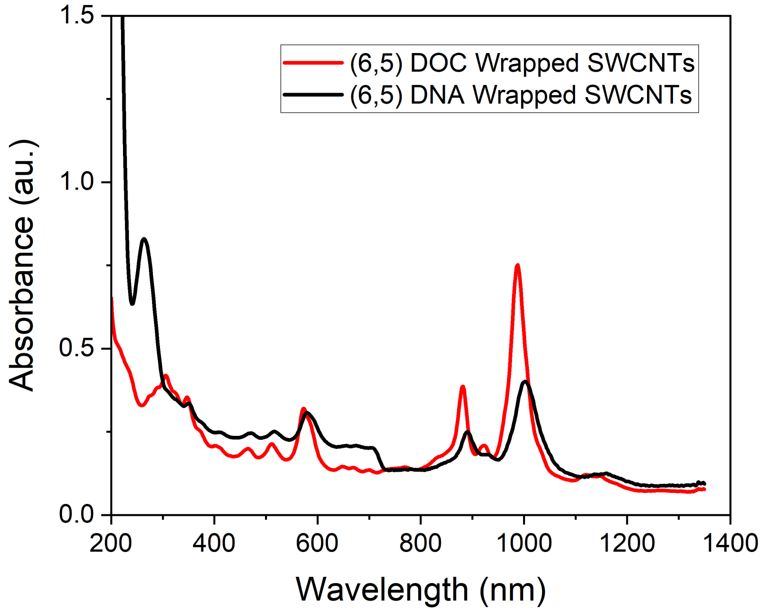

Figure S1. UV-Vis spectrum obtained from dispersed SWCNT samples displaying the red shift of the DOC wrapped SWCNTs to DNA wrapped SWCNTs that occurred through the DNA exchange procedure (1).

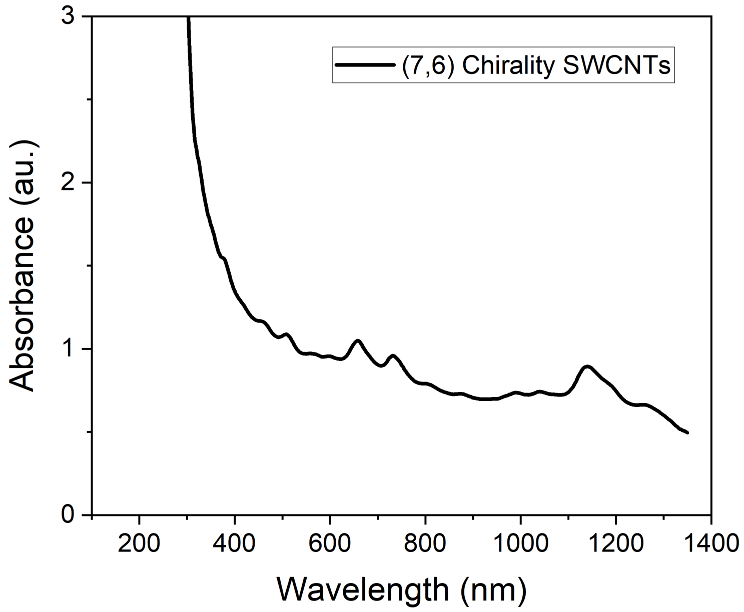

Figure S2. UV-Vis spectrum obtained from DNA wrapped (7,6) SWCNTs.

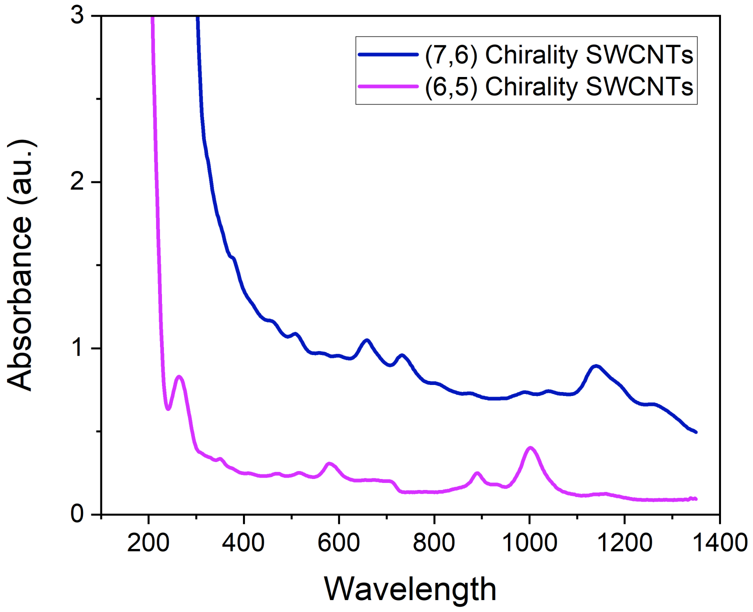

Figure S3. UV-Vis spectrum comparing the absorbance values for (6,5) and (7,6) DNA wrapped SWCNTs.

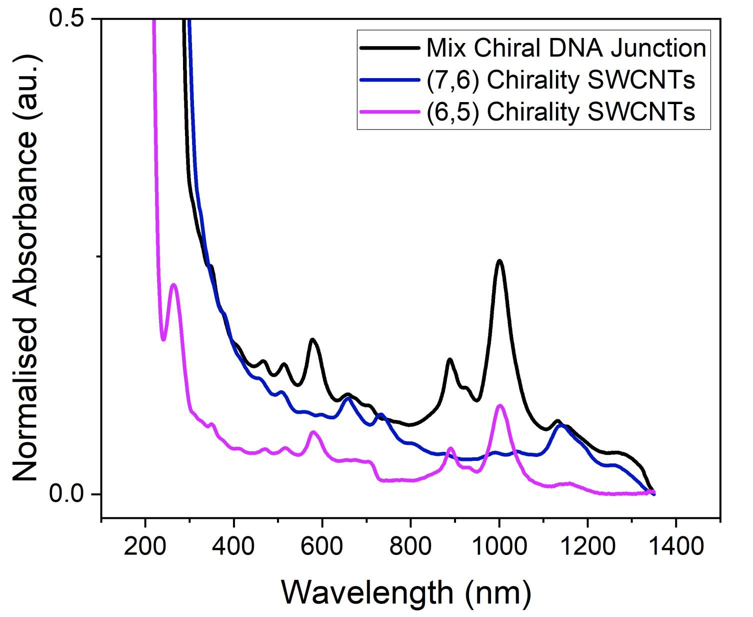

Figure S4. Normalised UV-Vis spectra comparing the absorbance values for (6,5) and (7,6) DNA wrapped SWCNTs with the Mix chirality junction.

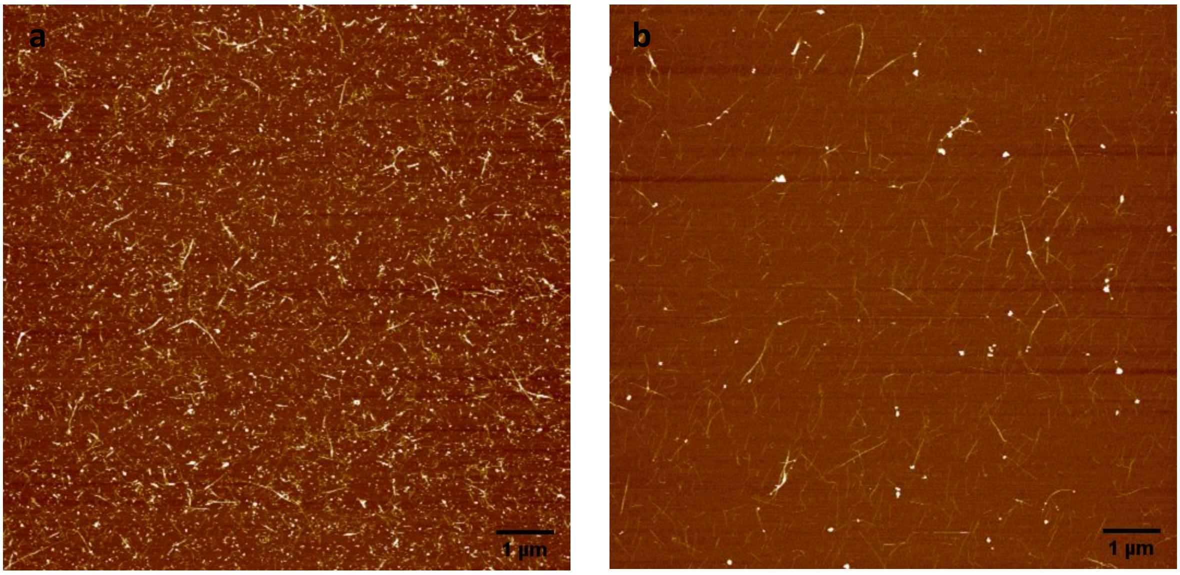

Figure S5. AFM Images of SWCNT solutions; a) (7,6) DNA wrapped SWCNTs; b) (6,5) DNA wrapped SWCNTs.

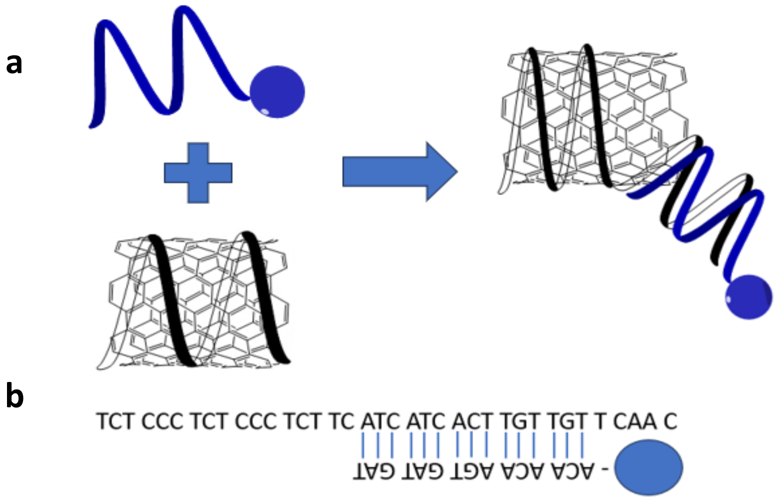

Scheme S1. a) A schematic of the hybridisation between the wrapped SWCNT and the streptavidin-conjugated biotinylated DNA; b) The sequences used with the DNA hybridisation shown, the blue particle indicates streptavidin.

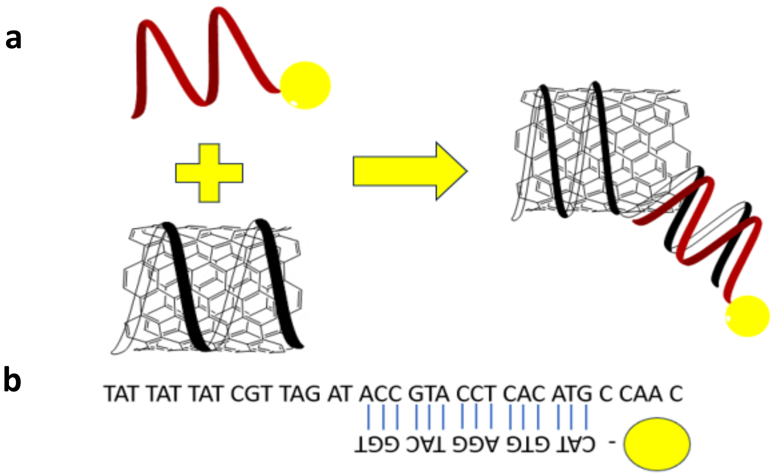

Scheme S2. a) A schematic of the hybridisation between the wrapped SWCNT and the AuNP-conjugated thiolated DNA; b) The sequences used with the DNA hybridisation shown, the yellow particle indicates AuNP.

a

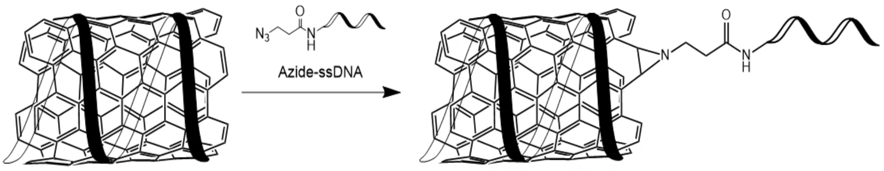

b

CCC GGG CCC GGG CCC GGG CCC

GGG CCC GGG CCC GGG CCC GGG

Scheme S3. a) Schematic of Azide-functionalised DNA reacting with SWCNTs (2); b) DNA strands used for the junction formation alongside how they hybridise with each other.

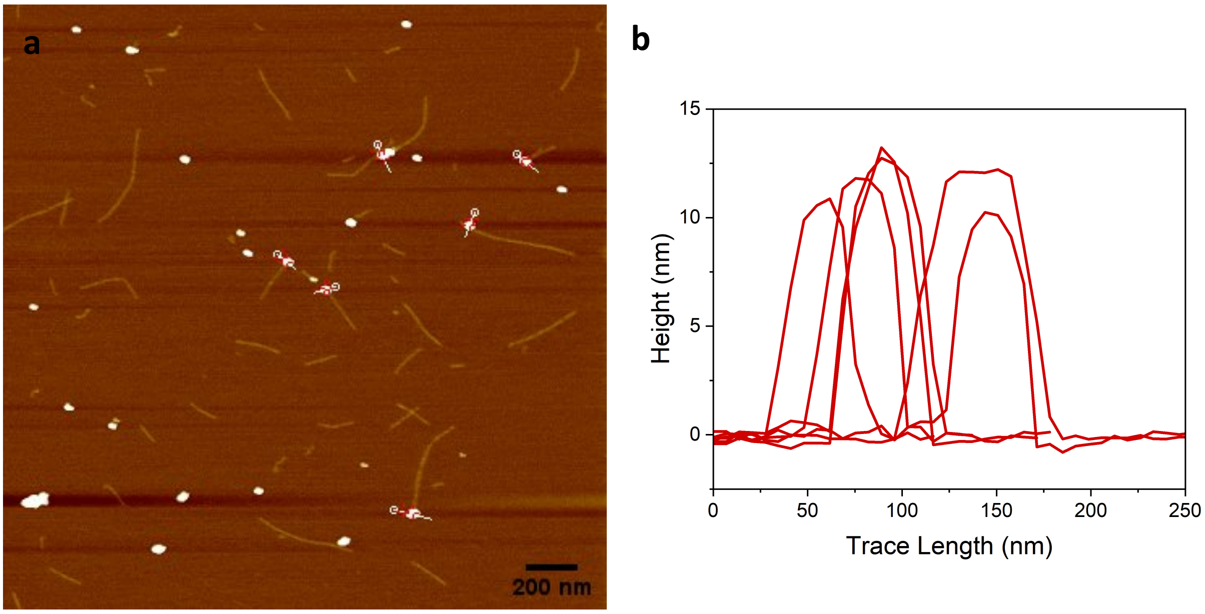

Figure S6. a) AuNP tagging of (6,5) DNA wrapped SWCNTs; b) Height profile of selected particles within the sample displaying heights within the range of 10-14nm.

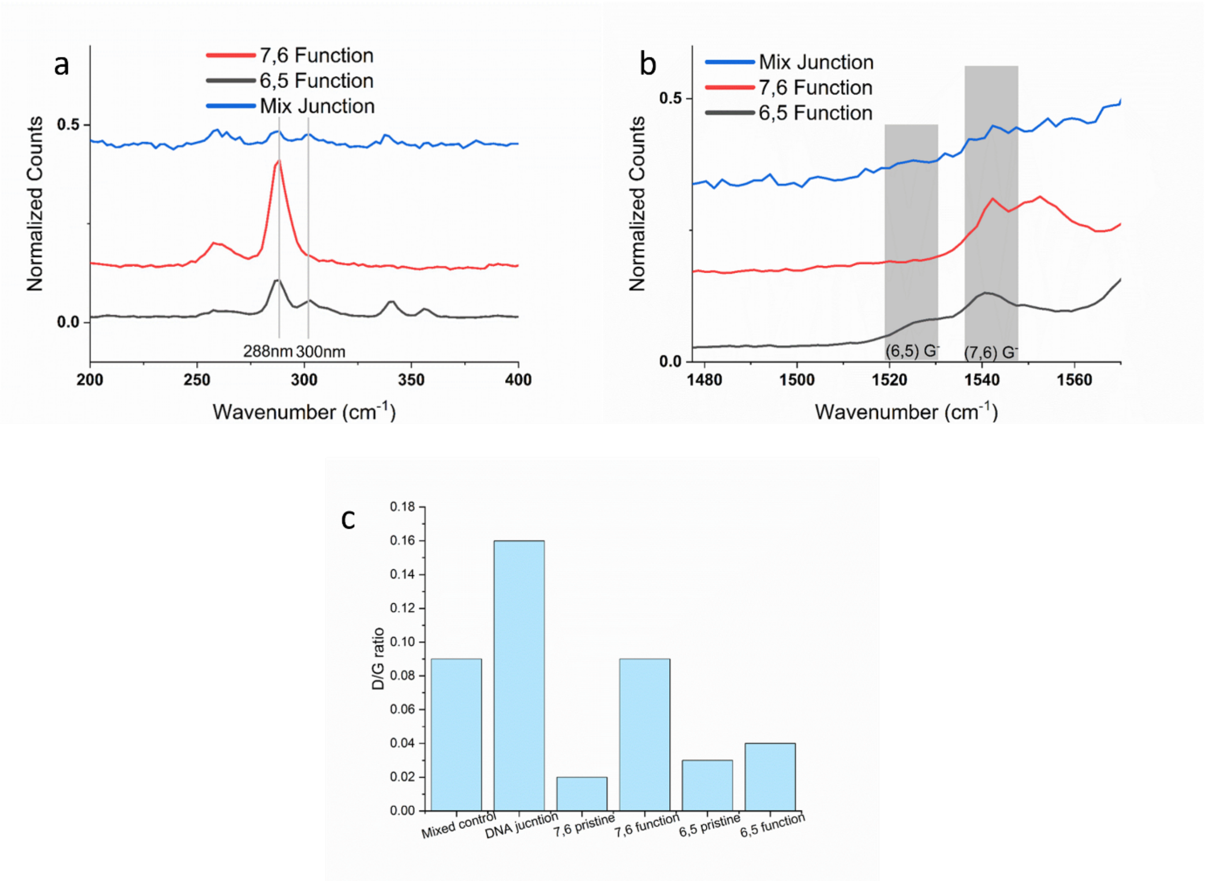

Figure S7. Raman Spectra of the functionalized carbon nanotubes and formed junction. a) RBM of the functionalized carbon nanotubes and the mix chirality junction solution; b) G Band spectra of the functionalized carbon nanotubes and the mix chirality junction; c) D/G ratio comparing the pristine material with their functionalized counterparts.

Figure S7 displays the Raman spectra obtained for the functionalized carbon nanotubes, both the RBM (Radial Breathing Mode) (3) and G-/G+ bands (4) provide us with chirality information. The spectra are consistent with the descriptions in the literature, the RBM peak at 288 cm^{-1} corresponds to the (7,6) chirality, while the peak at 300 cm^{-1} corresponds to the (6,5) chirality. As reported in the literature, although the (6,5) chirality is the major component of the sample, its response is weaker under 633 nm laser excitation. In the junction samples, the G- peaks for (6,5) at 1527 cm^{-1} and (7,6) at 1542 cm^{-1} are also observed. Additionally, an increase in the D/G ratio (5) is seen in the functionalized samples, which suggests successful functionalization.

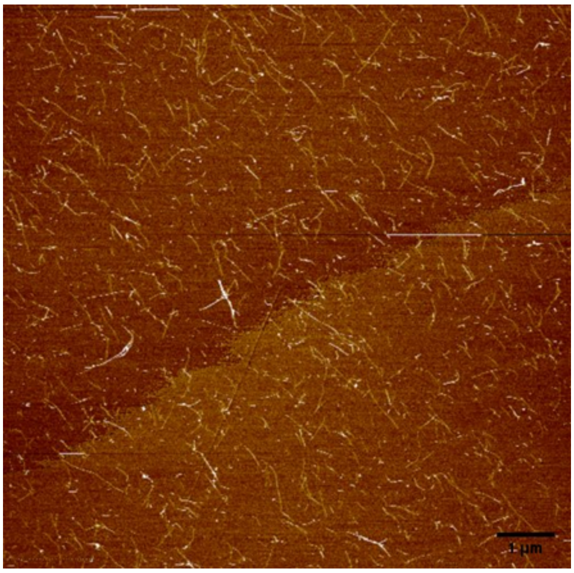

Figure S8. AFM image of (7,6) SWCNT-DNA-(6,5) SWCNT junction.

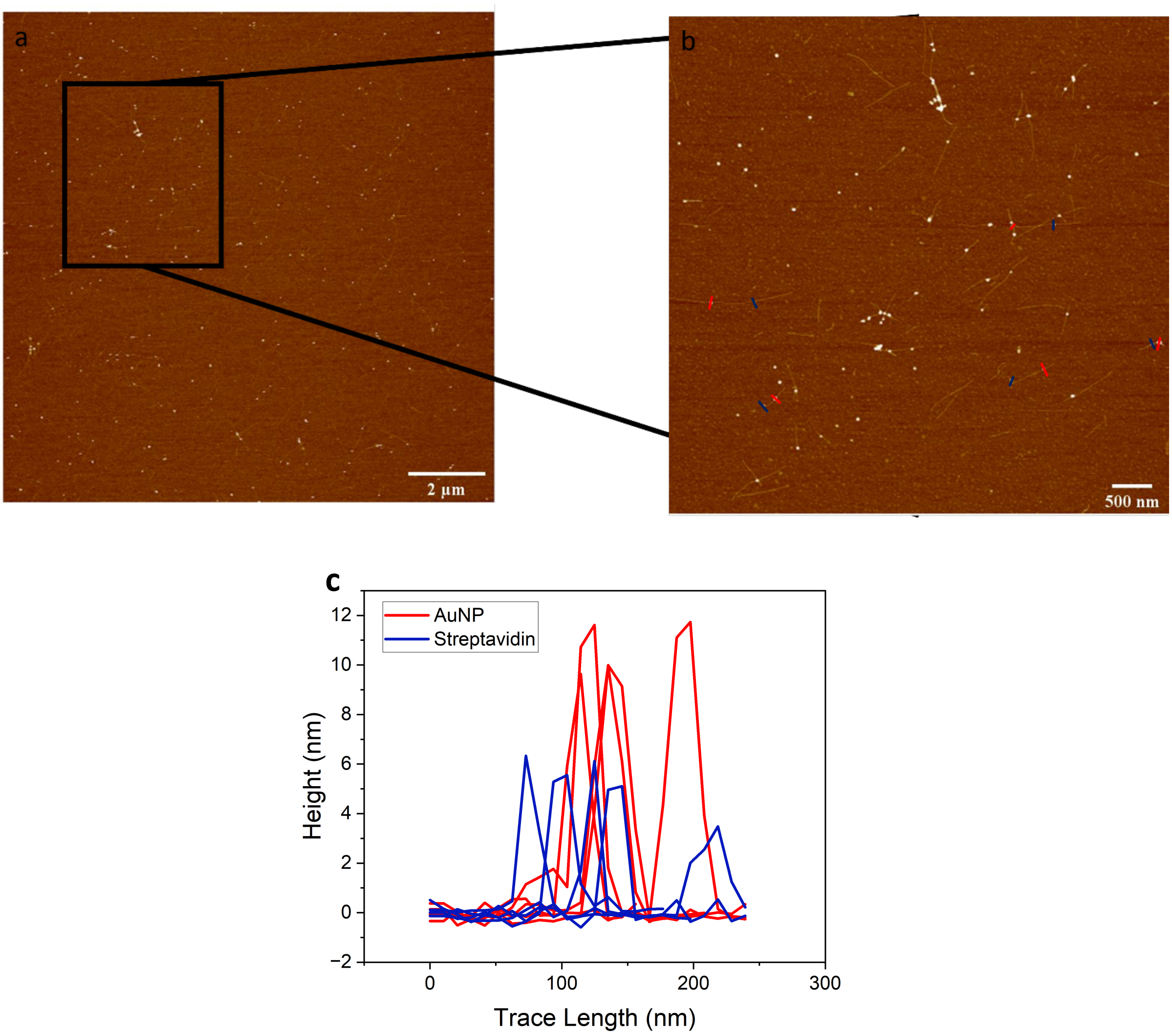

Figure S9 a) Large-scale AFM image of labelled mix chiral DNA junctions; b) Magnified area from the initial image; c) Height profiles of various particles tagged on the junctions, blue for streptavidin and red for AuNP.

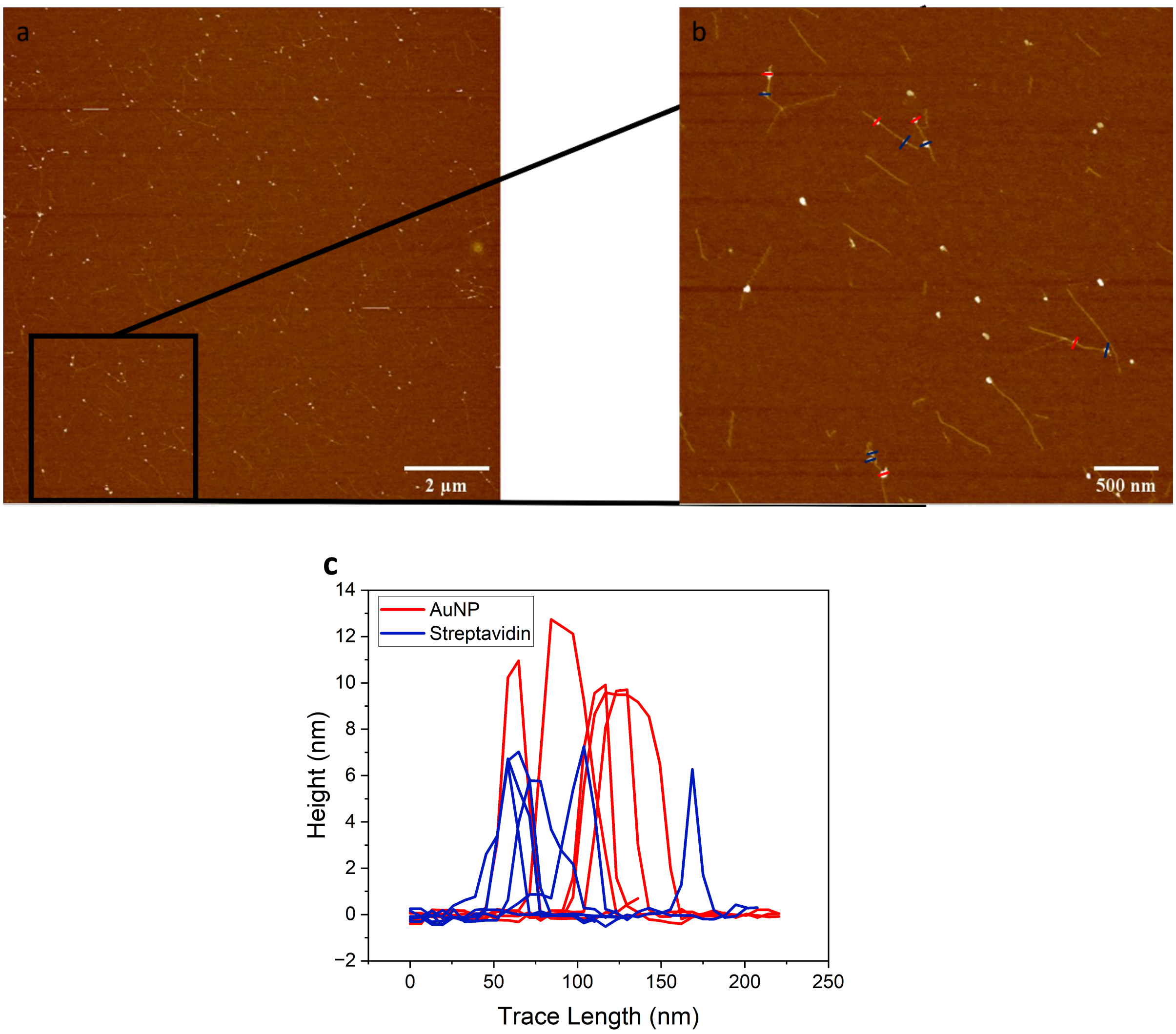

Figure S10 a) Large-scale AFM image of labelled mix chiral DNA junctions; b) Magnified area from the initial image; c) Height profiles of various particles tagged on the junctions, blue for streptavidin and red for AuNP.

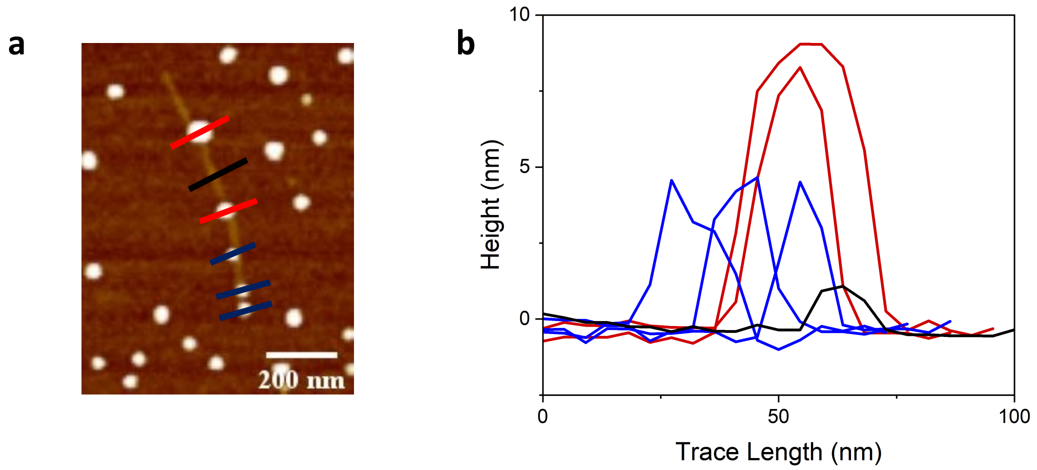

Figure S11. a) Double tagged SWCNT-DNA-SWNCT junction; b) Height profile displaying the two different tagging, with the dark red lines corresponding to the AuNP, blue lines to the streptavidin and black line to the SWCNT.

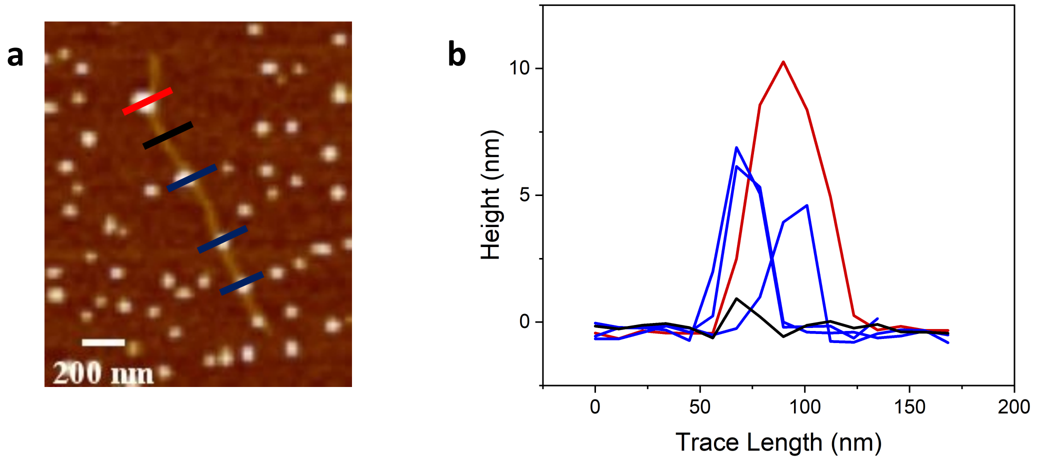

Figure S12. a) Double tagged SWCNT-DNA-SWNCT junction; b) Height profile displaying the two different tagging, with the dark red lines corresponding to the AuNP, blue lines to the streptavidin and the black line to the SWCNT.

References

(1): Streit, J.K.; Fagan, J.A.; Zheng, M. A low energy route to DNA-wrapped carbon nanotubes via replacement of bile salt surfactants. *Anal. Chem.* **2017**, 89 (19), 10496–10503. DOI: 10.1021/acs.analchem.7b02637

(2): Amoroso, G.; Ye, Q.; Cervantes-Salguero, K.; Fernández, G.; Cecconello, A.; Palma, M. DNA-Powered Stimuli-Responsive Single-Walled Carbon Nanotube Junctions. *Chem. Mater.* **2019**, 31 (5), 1537-1542. DOI: 10.1021/acs.chemmater.8b04483

(3): Tian, Y.; Jiang, H.; Laiho, P.; Kauppinen, E. L. Validity of measuring metallic and semiconducting single-walled carbon nanotube fractions by Quantitative Raman Spectroscopy. *Anal. Chem.* **2018**, 90 (4), 2517–2525. DOI: 10.1021/acs.analchem.7b03712

(4): Telg, H.; Duque, J. G.; Staiger, M.; Tu, X.; Hennrich, F.; Kappes, M. M.; Zheng, M.; Maultzsch, J.; Thomsen, C.; Dorn, S. K. Chiral index dependence of the g+ and g– raman modes in semiconducting carbon nanotubes. *ACS Nano* **2012**, 6 (1), 904–911. DOI: 10.1021/nn2044356

(5): Yan, X.; Itoh, T.; Kitahama, Y.; Suzuki, T.; Sato, H.; Miyake, T.; Ozaki, Y. A Raman spectroscopy study on single-wall carbon nanotube/polystyrene nanocomposites: Mechanical compression transferred from the polymer to single-wall carbon nanotubes. *J. Phys. Chem. C* **2012**, 116 (33) 17897–17903. DOI: 10.1021/jp303509g
